# Leaving no patient behind! Expert recommendation in the use of innovative technologies for diagnosing rare diseases

**DOI:** 10.1186/s13023-024-03361-0

**Published:** 2024-09-27

**Authors:** Clara D. M. van Karnebeek, Anne O’Donnell-Luria, Gareth Baynam, Anaïs Baudot, Tudor Groza, Judith J. M. Jans, Timo Lassmann, Mary Catherine V. Letinturier, Stephen B. Montgomery, Peter N. Robinson, Stefaan Sansen, Ruty Mehrian-Shai, Charles Steward, Kenjiro Kosaki, Patricia Durao, Bekim Sadikovic

**Affiliations:** 1 Departments of Pediatrics and Human Genetics, Emma Center for Personalized Medicine, Amsterdam Gastro-Enterology Endocrinology Metabolism, Amsterdam University Medical Centers, Amsterdam, The Netherlands; 2 Program in Medical and Population Genetics, Broad Institute of MIT and Harvard, Cambridge, USA; 3 Division of Genetics and Genomics, Boston Children’s Hospital, Boston, USA; 4 Rare Care Centre, Perth Children’s Hospital and Western Australian Register of Developmental Anomalies, King Edward Memorial Hospital, Perth, Australia; 5 Aix Marseille Univ, INSERM, Marseille Medical Genetics, MMG, Marseille, France; 6 European Molecular Biology Laboratory (EMBL-EBI), European Bioinformatics Institute, Hinxton, UK; 7 Department of Genetics, Section Metabolic Diagnostics, University Medical Center Utrecht, Utrecht, The Netherlands; 8 Telethon Kids Institute, Nedlands, Australia; 9 IRDiRC Scientific Secretariat, French National Institute of Health and Medical Research (INSERM), Paris, France; 10 Stanford University School of Medicine, Stanford, USA; 11 The Jackson Laboratory, Farmington, CT USA; 12 Sanofi, Diegem, Belgium; 13 Pediatric Brain Cancer Molecular Lab, Sheba Medical Center, Ramat Gan, Israel; 14 Genomics England, London, UK; 15 Keio University, Minato, Japan; 16 The Cure and Action for Tay-Sachs (CATS) Foundation, Altringham, UK; 17 Verspeeten Clinical Genome Centre, London Health Sciences, London, Canada; 18 Department of Pathology and Laboratory Medicine, Western University, London, Canada

**Keywords:** Rare disease, Rare disease diagnosis, Innovative technologies, IRDiRC, Rare disease research, Genomics, Molecular diagnostics

## Abstract

Genetic diagnosis plays a crucial role in rare diseases, particularly with the increasing availability of emerging and accessible treatments. The International Rare Diseases Research Consortium (IRDiRC) has set its primary goal as: “*Ensuring that all patients who present with a suspected rare disease receive a diagnosis within one year if their disorder is documented in the medical literature”*. Despite significant advances in genomic sequencing technologies, more than half of the patients with suspected Mendelian disorders remain undiagnosed. In response, IRDiRC proposes the establishment of “*a globally coordinated diagnostic and research pipeline*”. To help facilitate this, IRDiRC formed the Task Force on Integrating New Technologies for Rare Disease Diagnosis. This multi-stakeholder Task Force aims to provide an overview of the current state of innovative diagnostic technologies for clinicians and researchers, focusing on the patient’s diagnostic journey. Herein, we provide an overview of a broad spectrum of emerging diagnostic technologies involving genomics, epigenomics and multi-omics, functional testing and model systems, data sharing, bioinformatics, and Artificial Intelligence (AI), highlighting their advantages, limitations, and the current state of clinical adaption. We provide expert recommendations outlining the stepwise application of these innovative technologies in the diagnostic pathways while considering global differences in accessibility. The importance of FAIR (Findability, Accessibility, Interoperability, and Reusability) and CARE (Collective benefit, Authority to control, Responsibility, and Ethics) data management is emphasized, along with the need for enhanced and continuing education in medical genomics. We provide a perspective on future technological developments in genome diagnostics and their integration into clinical practice. Lastly, we summarize the challenges related to genomic diversity and accessibility, highlighting the significance of innovative diagnostic technologies, global collaboration, and equitable access to diagnosis and treatment for people living with rare disease.

## Introduction

Accurate diagnosis is a cornerstone of rare disease (RD) patient care; however, a significant proportion of patients with RD remains undiagnosed [1]. This is harmful as a diagnosis is essential for accurate genetic counselling, including recurrence risk in future children and identification of family members at risk, for information sharing and understanding. Receiving an accurate diagnosis might remove feelings of guilt, lead to better access to community services, and allow for personalized management, including targeted therapy and prevention [1]. The window of therapeutic opportunity is often missed if diagnosis is delayed or not achieved at all [2]. To optimize health outcomes for individuals with RDs, establishing a diagnosis should be prioritized in accordance with the International Rare Diseases Research Consortium’s (IRDiRC) goals: “All patients coming to medical attention with a suspected RD will be diagnosed within one year if their disorder is known in the medical literature; all currently undiagnosable individuals will enter a globally coordinated diagnostic and research pipeline” [2]. Although genomic sequencing technologies have revolutionized our ability to diagnose Mendelian diseases, at least half of all patients remain without a diagnosis [3]. Given this large unmet medical need on the one hand and the rapid technological evolution on the other, IRDiRC launched the Task Force on Integrating New Technologies for Rare Disease Diagnosis to draft an article to support the advancement of diagnostic and research pipelines around the globe. To this end, our Task Force, comprising relevant RD stakeholders, has reviewed the most recent advances in innovative diagnostic technologies for clinicians and researchers. Highlighting the patient’s diagnostic journey, we outline different technologies, their strengths and limitations, and their current state of use, exemplified by case vignettes. Herein, we provide expert recommendations to support the diagnostic process and enable access to personalized therapy and care.

Challenges related to genomic diversity and implications related to the global (in-)equity in access, are discussed. The importance of FAIR and CARE data management [3], as well as genomics education and training beyond academia is highlighted. We end with future technological developments and the transition of these technologies from research to clinical practice. We hope that our article will support the goal of ‘leaving no patient behind,’ and ultimately contribute to improving the diagnosis and care of RD patients globally.

## Methods

To develop this manuscript, we used a combination of expert opinion and literature review. The International Rare Diseases Research Consortium (IRDiRC) Task Force on Integrating New Technologies for Rare Disease Diagnosis is composed of renowned multi-stakeholder experts in RD diagnosis and research. The Task Force members were consulted to provide their expert opinions on the latest advances in innovative diagnostic technologies and their potential applications in the diagnosis of RDs.

In addition to collective expert opinion, we conducted a scoping literature review in September 2023 to identify the latest research and advancements in the field of RD diagnosis. We performed searches for relevant articles in PubMed, Google Scholar, ScienceOpen, and NIH National Library of Medicine, using keywords such as “rare disease diagnosis,” “genomics,” “multi-omics,” “functional testing,” “model systems,” “bioinformatics,” and “artificial intelligence.” We also reviewed relevant guidelines and recommendations from national and international RD organizations.

We assessed the advantages and limitations of each omics technology and the current state of utilization in clinical practice. We also explored the challenges associated with global differences in accessibility and proposed a stepwise approach to the application of these innovative technologies to support the diagnostic process and enable access to personalized therapy and care. The fact that the current review is scoping rather than systematic in nature poses a limitation; this is counterbalanced by the diversity in rare diseases expertise and backgrounds of the authors which enables a comprehensive and well-rounded overview of this topic.

The Task Force members also discussed the importance of FAIR data management, genomics education and training beyond academia, future technological developments, and the transition of these technologies from research to clinical practice. These factors were evaluated in light of the current lack of global equity of access to RD diagnosis and care.

### Key findings of the task force

#### Patient perspective—patients at the forefront

Patients are the most important advocates for their disorders, providing essential information e.g., through patient registries [4], supporting other people affected by the same disorder, raising the funding required to further research into therapeutics, and making disease-specific information available in a clear and easy to understand form. Consideration of making key publications freely available for patients and participants is therefore essential [5].

Patients and patient advocates can also bring clinicians and scientists together to support research by establishing scientific advisory boards. This is particularly important for RDs, where there are often only a handful of people identified globally with a given disorder. For these ultra-rare conditions, there is limited interest from the industry in developing therapies due to the perception of a lack of Return On Investment (ROI) and the challenges of creating a development program unless significant additional incentives such as the Orphan Medicinal Products Regulations are in place, and socially responsible frameworks for public–private partnerships are developed and used [6]. More research is also required to understand the impact of an early diagnosis in RD versus one later in life.

Clinical trials often struggle to identify and enroll sufficient patients, and this could potentially be solved if effective, accurate, and patient-centric testing is widely available to increase diagnosis. The Global Alliance for Genomics and Health (GA4GH) is promoting the concept of a global genomic database federation to allow the secure sharing of genomic and healthcare data, increasing the probability of finding such rare patients [7]. Therefore, the key to using any innovative technology in diagnosing RDs is to have a patient-centric approach. All too often, the patient experience is either unaccounted for or considered too late in the design, which can cause delays or even the failure of initiatives [8]. An exemplar for patient involvement is the Participant Panel at Genomics England, which oversees what Genomics England and its partners do with their data [9]. The patient and participant are essential in many aspects of genomics/biotech and are central in planning research, advising on ethical issues, and aiding clinical trial readiness by having an organized and engaged patient community.

#### Innovative technologies for rare diseases

##### Deep phenotyping and reverse phenotyping

Deep phenotyping of patients suspected of having or diagnosed with a RD, optimally in a categoric and computer-readable format, has become standard practice in the last ten years. The Human Phenotype Ontology (HPO) [10] provides the most comprehensive resource for computational deep phenotyping and has become the de facto industry standard, used in analysis of exome and genome sequencing data [11, 12], as well as data integration in translational research and bioinformatics [13]. The ontology, maintained by the Monarch Initiative [14], provides a set of more than 15,500 terms describing human phenotypic abnormalities—arranged as a hierarchy. More than half of these terms have a plain language representation, allowing patients and families to become more effective partners in translational research [15]. Recently, translations into seven languages were made available.

Conversion of phenotype risk scores from the electronic health records to HPO terms enables differentiation of patients with Mendelian diseases from unaffected controls and assists in rare variant interpretation [16] or causative gene association [17]. Importantly, HPO data can be shared across platforms through the Phenopacket Schema, developed by GA4GH [18].

Reverse phenotyping is an approach in which specific clinical features are interrogated in a subsequent clinical examination based on the candidate genetic variants identified. It has been shown to increase RD diagnostic rate, particularly for disorders with high genetic heterogeneity and phenotypic complexity [19, 20], supporting the rationale for detailed clinical characterization at various stages of the diagnostic odyssey.

Where possible, deep phenotyping should be prospective (to the molecular test), objective to reduce (e.g., cognitive) bias, easily accessible (e.g., free and open access), and scalable for diversity, equity, and inclusion. However, when an expected phenotype is not reported, its absence should be confirmed through reverse phenotyping, as features that are part of the diagnosis may be overlooked or assumed to be familial.

### Genomics

Genome Sequencing (GS) applies Next Generation Sequencing (NGS) to assess three billion bases of human DNA, while Exome Sequencing (ES) focuses on the ~ 2% of protein-coding DNA (including ~ 19,000 genes), enabling a more focused, interpretable, and lower cost, albeit less comprehensive approach. Further focused approaches target known disease-causing genes (Mendeliome or Clinome, typically defined by Online Inheritance in Man (OMIM) [21] or the Gene Curation Coalition (GenCC) [22]). However, ES excludes noncoding and copy-neutral structural variation, may have limited sensitivity in complex genomic regions such as high CG density sequences, and is limited to pre-defined gene transcript isoforms. The diagnostic yield of ES is reported to average 40% for intellectual disability [23] and immunologic conditions [24] but may be higher for more specific phenotypes such as metabolic [25], neuromuscular [26], vision loss, and sensory deficits [27]. Currently, GS increases this diagnostic yield over ES by < 10% [28]. This is likely to increase as continuing advancements in analytic technologies and gene-disease associations advance, highlighting the need for reanalysis of preexisting patient NGS data, as has already been demonstrated [29, 30].

Resources for RD definitions, gene mappings, and ontologies that enable NGS analysis and interpretation include Orphanet [31], UMLS [32], MonDO [33], and OMIM [21], and tools that aggregate data from these resources [34]. Selection of potential variants and elucidation of the genetic basis of the disease is done by filtration and prioritization of variants, with higher diagnostic yields via trio- or family-based analysis given the ability to phase variants and identify de novo variation [35]. In addition, when available, a genomic medicine team review, phenotypic validation, and functional assessment, are important to eliminate the semi-automated NGS analysis that may miss 15% of diagnosis and 4% of candidate disease-causing variants [36]. With the reduction in sequencing costs, advancement of analysis tools, and increasing accumulations of databases, it’s likely that GS will be a feasible tool for diagnosing RDs.

### Long read sequencing

Advances in sequencing technologies have further expanded our ability to sequence from hundreds of base pairs with short read sequencing to tens of thousands of base pairs and occasionally millions of base pairs with long read sequencing [37]. These long reads provide improvements in the calling of short variants (SNVs and indels), particularly for genes with high homology with other regions of the genome, due to segmental duplications (i.e. *CBS*), paralogues (i.e. SMN1), or highly homologous gene families. Additionally, about three times as many structural variants are identifiable from long read sequencing, and there is improved resolution of tandem repeats. Haplotype phasing can support evaluation for compound heterozygous variant pairs without requiring DNA from the parents. DNA methylation can also be detected by this technology, which may be applicable to episignature assessment. Due to the need for high molecular weight DNA, analytic challenges including the lack of reference data, and higher costs, access to long read genome sequencing is currently more in research settings.

Several proof-of-principle studies have used long read sequencing technologies for RD diagnosis [38, 39]. Thus far, the majority of the genetic diagnoses achieved are also detectable by short read sequencing. For variants exclusively detectible by long read sequencing, it is challenging to differentiate between common from the rare (and ultra-rare) variants, further limiting the clinical utility of this approach at the moment [40, 41]. Applications of this technology in single cell and bulk RNA sequencing can enable gene transcript differentiation, which holds potential for use in RD diagnosis [42]. Another successful application of this technology involves targeted sequencing of genes strongly related to a patient’s phenotype without variants detectable by conventional analyses. The diagnostic power of long read sequencing is expected to increase as analysis approaches and reference data are developed, and this remains a promising area for increasing diagnosis.

### DNA methylation (DNAm) episignatures

The classic definition of epigenetics is mitotically heritable changes in gene expression without alteration of the DNA sequence and includes DNA methylation at CpG motifs [43]. However, an increasing number and spectrum of rare disorders exhibit the so-called DNA methylation episignatures, defined as recurring, sensitive, and specific DNA methylation biomarkers associated with a common genetic or environmental etiology [44]. DNA methylation episignatures can be used to help resolve ambiguous clinical and genetic findings, including genetic variants of unknown significance, and evaluate undiagnosed patients with RDs [45, 46].

Episignatures are developed by computational machine learning models primarily using methylation microarray data from peripheral blood samples in cohorts of individuals with common genetic or environmental etiology. The ability to detect episignatures is contingent upon the intensity (effect size) and extent (number of differentially methylated CpGs) of the observed DNAm changes, which can range from tens of thousands of differentially methylated CpGs (Sotos and NSD1, Tatton-Brown-Rahman syndrome and DNMT3A) to only a few hundred (BAFopathies) [46, 47]. The presence of an episignature is considered strong functional evidence that can aid in the reclassification of VUSs to a likely pathogenic status. In contrast, the absence of an episignature is considered supportive but not definitive for lack of pathogenicity [48]. Current limitations of this technology may include technical batch effects of microarray analysis, limited detection in mosaic cases, still limited (but growing) number of RDs with the defined episignatures, and a limited number of clinical diagnostic laboratories providing this testing.

Application of this technology to the broader patient populations will depend on the rate of discovery of gene and disorder-specific episignatures. Larger-scale studies are necessary to assess the diagnostic yield and health system impact as either a first-line test or in unresolved cases post-genomic assessment. Finally, the development of clinical recommendations and guidelines for the use and application of DNA methylation episignature analysis is warranted and currently ongoing.

### Data sharing

Data sharing initiatives have greatly enhanced our understanding of genetic variation in human populations and RDs. As new gene-disease relationships (GDR) are discovered and published in the literature, they are reviewed and added to databases like OMIM. This consideration applies to diagnostic laboratories and collaborative research initiatives like Clinical Genome Resource (ClinGen), Genomics England PanelApp, and PanelApp Australia, and are now shared through the Gene Curation Coalition (GenCC) database [22]. The ClinVar database specializes in collecting variant interpretations by clinical testing laboratories along with other submitters [49].

Many genetic causes of RD remain to be identified [50]; most RDs are exceedingly rare, often with a prevalence of < 1 in 1–100 million individuals. Individual systems were developed to support two-sided gene matching–between researchers or clinicians with the same gene candidate. Further advancements came from the launch of the Matchmaker Exchange (MME) in 2015, a federated network connecting multiple databases through a common application programming interface (API). Already, MME has > 13,000 unique genes from > 120,000 cases submitted by > 12,000 contributors in 98 countries across eight matchmaking nodes [51]. About half of the gene submissions receive a match, and approximately 15% of matches are “successful”, which is determined through follow-up email exchanges. Hundreds of gene-disease discoveries have been made through the MME through the engagement of large research initiatives, independent researchers and clinicians, and clinical testing laboratories [52–55].

Another type of matchmaking involves early efforts to support variant matching—to allow querying of sequenced datasets for specific variants or classes of variants (i.e. loss of function variants) in a candidate gene [56]. Several databases with this functionality exist today (i.e. VariantMatcher, Franklin, Geno2MP), but they are not yet connected through the MME, and the amount of data currently queryable in these systems is limited.

Meaningful data sharing requires adhering to the FAIR principles [57]. Data should be shared in databases that are well known in the RD clinical and research communities, be accessible through controlled access mechanisms that protect research participants, be well organized and structured so those who access the data understand it, with a sufficient amount of information (i.e., detailed phenotype) to support independent analysis. Federated systems such as MME provide a good model where the data can be locally hosted while providing global access to allow the use of the data to improve gene discovery and diagnosis. Cloud-based research environments like AnVIL [58] and Genomics England provide secure environments where the tools can be brought to the data to support research.

### RNA sequencing and transcriptome datasets

RNA sequencing has increasingly emerged as a tool for RD diagnosis [59]. It enables the detection of aberrant gene expression, splicing, or allelic expression that can be paired with NGS of DNA to focus analysis onto genes with altered transcription, or it can be used to aid in interpreting VUSs identified through ES/GS analysis [60–62]. Studies have reported diagnostic yields ranging from 7 to 36% [63, 64]. Challenges with this approach which need further work relate to the temporal (developmental stage) and spatial (tissue type) variability in gene expression and limited analytical pipelines.

Reduced sequencing costs have driven the creation of comprehensive functional genomic atlases, including FANTOM [65], ENCODE [66], GTEx [67], and the Human Cell Atlas [68], which catalogue genes and their expression levels across different tissues and organs. The rapid progress made in identifying RD variants now enables the systematic exploration of associations between RD phenotypes and tissue and cell types. Shared patterns of gene expression linked to phenotypes can be used to evaluate the impact of genetic variants while furthering our understanding of disease etiology by defining the affected cell types and developmental time windows.

### Additional OMICS approaches

Several additional omics strategies are developed and, to varying extents, implemented in clinical practice to pursue optimal diagnostic coverage for patients with RD. These include ATAC-sequencing, metabolomics, and lipidomics. Since the individual-omics platforms address different aspects of (patho)physiology, they may increase the diagnostic yield. Multiple diagnostic technologies may be applied simultaneously or sequentially (e.g. metabolomics to follow up on a VUS identified by sequencing) [1].

#### ATAC-seq

Gene expression is directly related to chromatin accessibility. An increasing number of RDs involve genes that impact chromatin accessibility [69, 70]. Assay for Transposase-Accessible Chromatin using sequencing (ATAC-seq) assesses chromatin accessibility and, as such, identifies active (i.e. euchromatin) and inactive (i.e. heterochromatin) regions of the genome. Currently, the diagnostic application of ATAC-seq is limited as it is mostly applied in research settings [71]. Ongoing efforts are directed towards methodological improvements of ATAC-seq for multiple cell types and tissues, with the goal of diagnostic utilization [72].

#### Metabolomics

Metabolites, small organic molecules, are intermediates or end products of enzymatic processes and, as such, faithfully reflect ongoing (patho)physiological processes. Their levels in biological fluids such as blood or urine may vary based on gene function, disease processes, and exogenous factors (diet, environment, medication). Traditionally, metabolic diagnostics are performed in a targeted manner, assessing levels of specific metabolites, and guided by phenotypic or molecular findings. Recent technological advances have led to the introduction of untargeted metabolomics, a considerably more comprehensive test acquiring a near-complete view of the metabolome, in diagnostic practice [73]. As expected, the diagnostic yield of untargeted metabolomics is significantly increased when compared to conventional metabolic screening [74]. However, the increased diagnostic yield for patients seeking a diagnosis after the initial evaluation is more limited [75]. Although not accessible yet in the large majority of clinical centers, untargeted metabolomics is now positioned in the early clinical stages of the diagnostic arena and is becoming the standard of care in some centers.

#### Lipidomics

Compared to metabolomics, the diagnostic implementation of untargeted analysis of lipids, i.e. lipidomics, is in its infancy due to the biochemical complexity of this class of molecules and the relatively limited clinical evidence. Parallel to metabolomics, however, technological developments in mass spectrometry have significantly advanced the possibilities of lipidomic studies in biological fluids and patients’ cells. Lipidomics is currently not applied in RD diagnostics of individual patients, but there are ongoing research efforts in developing reference datasets in patient cohorts and controls. There have been advancements involving lipid biomarkers and understanding the underlying pathophysiology of lipid metabolism disorders, such as peroxisomal disorders [76], which may enable clinical adoption in the coming years.

### High throughput functional studies

Multiplexed assays for variant effects (MAVEs) provide new opportunities to test and prioritize rare genetic variants *en masse* [77–79]. A notable example of this approach has enabled profiling all possible SNVs, including missense variants for functional effects in critical regions of *BRCA1* [80]. Comparable applications have since been extended to map functional effects for missense variants for genes such as *MSH2* in Lynch Syndrome [81], *KCNH2* in long QT syndrome [82], and *NPC1* in Niemann-Pick disease type C [83]. In addition, several massively parallel assays enable more routine testing of the functional effects of variants in non-coding regions, including regulatory regions, splice junctions, and UTR sequences [84–86]. Albeit limited to the specific genes, these data are improving computational approaches for classifying rare variants for pathogenicity [87, 88] and in the future, may facilitate RD diagnosis through the accessibility of an Atlas of Variant Effects (AVE) for many RD-relevant genes and regulatory regions [89]. Caution is warranted in the interpretation of non-coding variation without functional validation. Data sharing and collaborative efforts are required for expanding our understanding and assigning correct diagnoses in such clinical situations and phenotypes.

### Multi-OMICS

Several studies have shown complementarity and synergism in combining multiple omics modalities. For example, combining GS with ATAC-seq has led to novel associations of genes with disease [90]. In addition, integrative analyses of RNA-seq with ATAC-seq have revealed a novel marker in breast cancer [91]. A clear association between genetic variants and levels of metabolites in individual patients was recently described in a large study combining genomics and metabolomics [91], highlighting the metabolic and the underlying genetic diversity of humans and pinpointing genetic changes at and near gene loci that cause inherited metabolic disorders. The overall diagnostic efficiency of metabolomics and its specific benefits of genetic variants’ prioritization tool are currently under study; until then metabolomics has yet to be part of standard clinical care [92, 93].

### Model systems and organisms

Model systems have proven instrumental for understanding pathophysiology, confirming causal genotype–phenotype associations, and developing therapies for RD since studying the natural course of the disease in humans is limited due to low disease prevalence [94]. Model systems provide a powerful tool to understand the impact of genetic variation on phenotype and may include cultured cells (including primary, immortalized, or reprogrammed stem cell lines), organoids, yeast, worms, flies, fish, mice, or larger animals. Collaborative efforts are well underway to characterize knockout mice lines for every gene through the International Mouse Phenotyping Consortium [95]. Recapitulation of disease features and course—both clinical and biochemical—remains challenging. For example, Montoro and colleagues reviewed existing model systems for the neurometabolic disorder X-linked adrenoleukodystrophy (ALD) [96]. Model systems, ranging from cultured cells to plants to chimpanzees, share the genetic defect and biochemical aberrations associated with ALD, but each failed to fully recapitulate the disease, highlighting the challenge of choosing or creating an appropriate (human) disease model.

Using animal models as a diagnostic platform has evolved to a lesser extent than patient-derived material. Variants can be assessed in models by whole gene replacement or generation of specific variants. Each model system has both advantages and pitfalls, the choice of a model will depend on accessibility, ease of use, and, importantly, the presence of a valid readout for pathogenicity. While some genes may specifically affect a single cell type, others may cause systemic disease and may benefit from a whole animal model system. An additional challenge is mapping the human variation correctly into the genome of another organism. Recent advances in genome editing will aid in confirming diagnoses and developing model systems for RD.

Rapid evaluation of pathogenicity is essential for clinical utilization, and generating animal models with patient-specific variants is time-consuming. An alternate approach to changing the genome of the model system by introducing the variant of interest involves (over)expression of the patients’ gene in a knock-out rescue model system. Depending on the gene, model, and readout, these complementation assays may be done in transiently and relatively rapidly, enabling use in clinical diagnostics [97]. Recently, *C. elegans* whole gene humanized animal models were developed [98]. Increasing the availability of models will allow assessments of the functional consequences of many variants and will enhance the application of model systems in routine diagnostic care.

Combining gene editing of induced pluripotent stem cells (iPSCs) and cellular differentiation with transcriptomics is a powerful tool for studying RDs. iPSCs can be generated from easily accessible somatic cells like skin or blood and used to model the disease in vitro. Alternatively, CRISPR/Cas9 gene-editing technology allows the insertion of candidate variants into healthy iPSCs to create patient-specific disease models, whose effect can be assessed by RNA sequencing in the specific differentiated cell types. Bioinformatics tools, such as gene set enrichment analysis (GSEA), reveal perturbed pathways, known disease genes, and genes associated with particular HPO terms and allow for direct comparison to the patient’s phenotype [64]. Once established, these cellular disease models allow for further studies using molecular assays to understand disease etiology and develop new therapies.

### Artificial intelligence (AI)

Large-scale and heterogeneous data, from omics to medical records to images, are generated at an increasing pace on patients with RD. It can be difficult or impossible to manually interpret and integrate the data or develop rules for predicting the diagnosis or the response to treatment. AI approaches are particularly attractive in this context [99]. AI is an umbrella term encompassing both symbolic approaches, which explicitly represent and interrogate expert knowledge with rules, and numeric approaches (usually referred to as Machine Learning), which use algorithms to extract information from data automatically. Challenges include the lack of data for many RDs, the high degree of clinical variability within and across RDs, batch effects, and the many different data sources (DNA sequencing, clinical features, imaging, metabolic analysis, etc.), each of which requires different computational processing.

AI has been widely used for image analysis. Approximately 1 in 3 RDs have a facial phenotype, and these phenotypes are increasingly being refined in a 3D space due to advances in computer science and increasing accessibility of 3D imaging devices at reducing cost. 3D imaging overcomes the inherent limitations of 2D imaging. Also, 3D facial phenotypes can be converted to standard and computer-readable text outputs for integration with other text-based results and various omics technologies [100]. 3D imaging can also be used to monitor treatment and clinical trial response—demonstrating it as a technology that can bridge diagnosis to therapy [101].

AI approaches are also widely used for the analysis of omics data in general, the interpretation of genomic variants, and the integration of various types of multiomics and even multimodal data. Importantly, analysis and interpretation of biomedical data using AI approaches benefit from expert/prior knowledge. Symbolic AI is based on this principle and leverages Knowledge Representation (KR), representing information about a domain in a form that computer algorithms can use to solve complex tasks. Ontologies are an approach to KR widely used in RD research and clinical care. The previously described HPO, together with HPO-based computational models of over 8500 diseases, can enable specific weighted fuzzy matching between patient features and the disease models to enable clinical decision support [102, 103]. Approaches that extend this to prioritize genes and variants identified by sequencing have been shown to improve diagnostic pipelines by many projects, including the 100,000 Genomes project [104]. Aside from potential benefits, there are also risks in the application of AI. For example, when used by clinicians not exhaustively formed in molecular genetics, misdiagnoses might be made.

### How can these innovative technologies be applied efficiently to obtain a diagnosis & treatment and care?

**Fig. 1:**
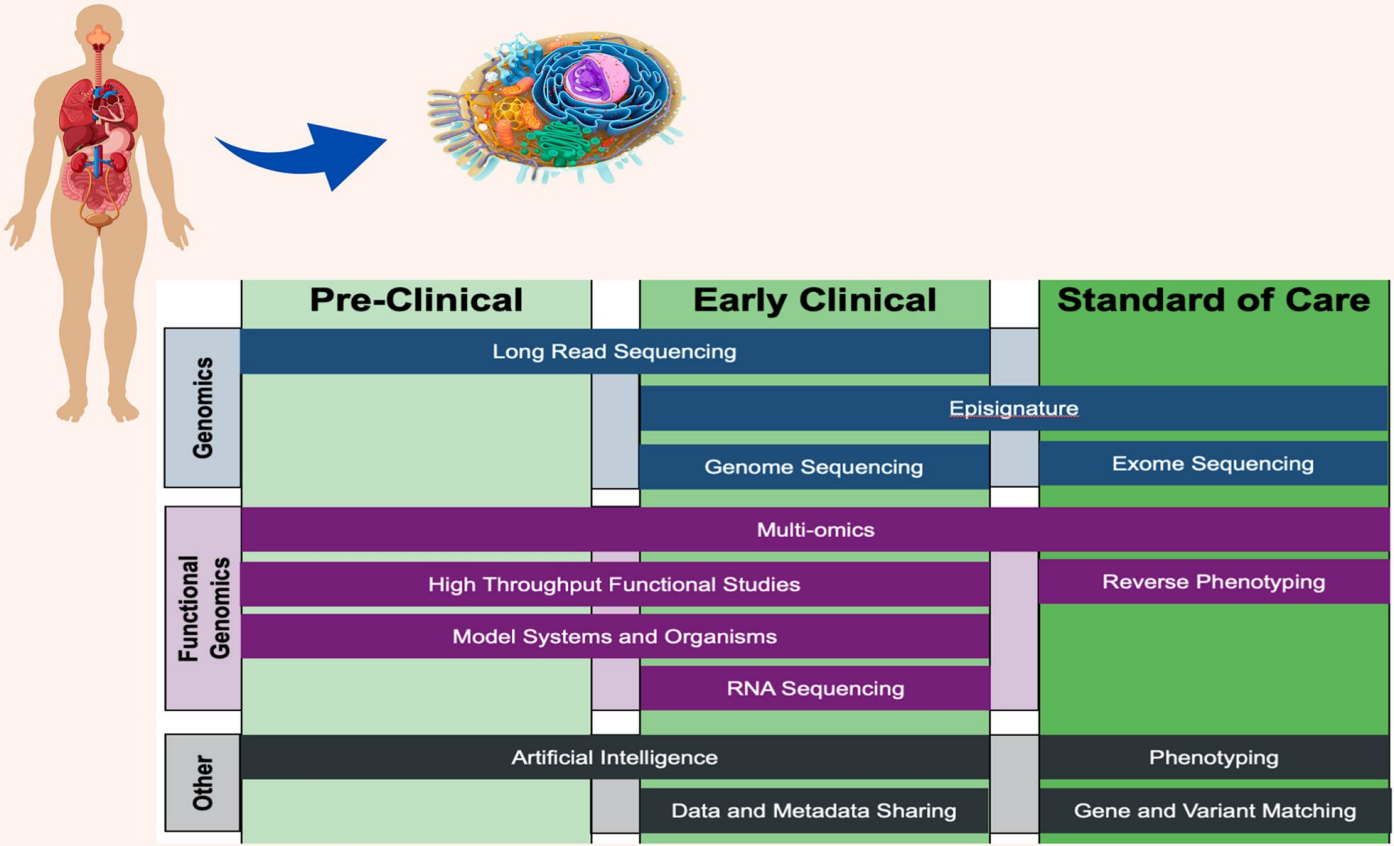
Innovative technologies enabling RD diagnosis and their current state of development

Broad application of novel technologies in clinical diagnostics commonly involves a progression through three stages: ‘pre-clinical’ (basic research), ‘early clinical’ (translational research, and ‘standard of care’ (clinical adoption) as illustrated in Fig. 1. The basic research stage normally involves the primary discovery and development of fundamental principles related to the technology. This typically happens in the basic academic laboratory setting and may provide some general insights into the potential application of the technology in the clinical setting, leading to the second stage of implementation involving translational research. At this stage, technology is systematically tested in relation to a particular clinical application. This involves typically larger clinical cohorts, consented through research protocols, and often involves clinical in addition to basic research laboratories. These studies can sometimes be national or international in scale and often aim to collect information about overall health systems impact in addition to validating the clinical utility of the technology. The final stage of clinical implementation of technologies that are ultimately proven valuable in the first two stages involves health systems implementation and clinical guidelines development. This stage can be highly variable globally, as it often depends on the jurisdictional health regulatory and funding implications. Jurisdictional health regulation is often national but can also be highly specific at the state/provincial level. Funding can also involve national or lesser jurisdictions such as health ministries and national health insurance, private health insurance, and patient pay systems, all adding layers of complexity. This final stage of implementation is often the longest and most challenging as it involves a much broader range of stakeholders, and it is often where promising technologies fail clinical adoption and implementation.

**Fig. 2:**
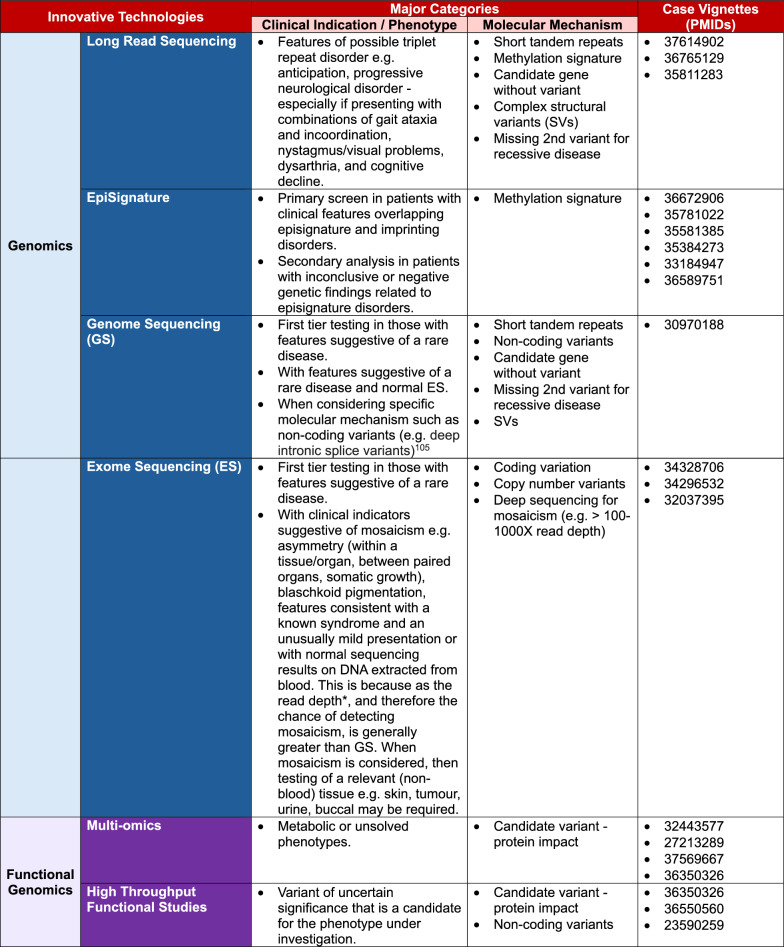

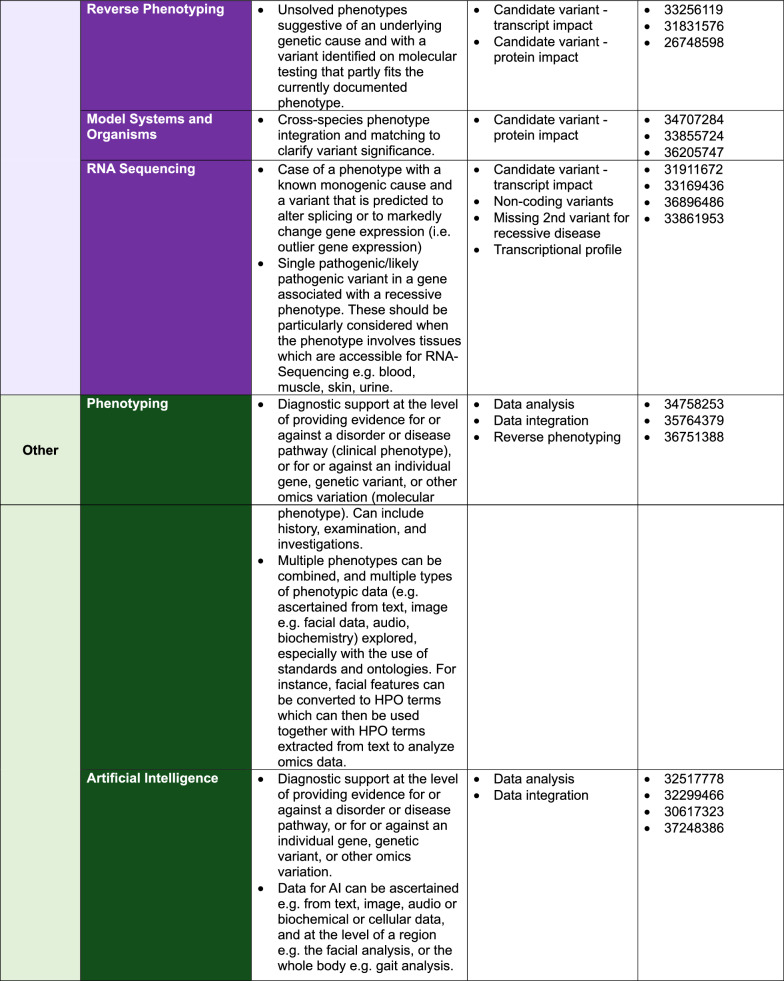

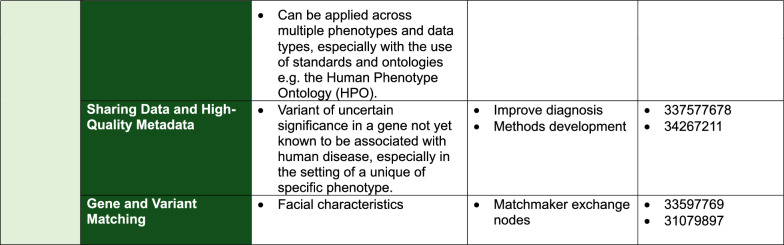
Matrix of innovative technologies and related phenotypic and molecular categories, including PMIDs of case vignettes. *Read depth: The number of times each individual base has been sequenced

Phenotypes and known/suspected molecular mechanisms can help guide the use of existing and novel technologies [29]. We provide expert recommendations via the decision matrix in Fig. 2 with factors to be considered and with reference to published case vignettes.

### From diagnosis to personalized therapy and care

A confirmed diagnosis is essential for closure, proper disease and prognostic information for the individual and family members, including genetic counselling for other family members at risk and future offspring, access to services in the community, and increasingly for therapeutic and preventive interventions. Knowing the cause of disease is a stepping stone for P4 medicine: Personalized, Predictive, Participatory, and Preventive [106].

Ideally, early diagnosis allows disease-modifying therapies to exert their effect in the crucial “neurodevelopmental time window,” potentially preventing (progression of) RD phenotypes [107] as well as somatic complications. Therapies targeting the underlying cause of disease include medical diet, nutritional supplements, (repurposed) pharmacologic medication, organ or stem cell transplantation, and increasingly regenerative and RNA/gene therapies. Supportive interventions such as physio- and speech therapy, and special aid at school are also essential for optimizing patient outcomes. Customized preventive measures, such as screening for malignancies or sick day protocols for inherited metabolic disorders, can also be taken once the diagnosis is known. All in all, management changes based on ES/GS diagnoses vary from 15 to 40% in the literature [108].

Care Pathways (CPWs) are required to structure and harmonize care processes and continuously improve them within the patient-centered care concept [109]. CPWs aim to have “the right person, in the right place, doing the right thing, at the right time, with the right outcome, and all with attention to the patient experience.” Evidence-based resources and CPWs may be developed for single RDs (e.g., Huntington’s disease [110]) or disorders encompassing multiple related etiologies (e.g., hereditary ataxias [111]). Steps or constituents of every CPW include various combinations of diagnostic procedures, specific or symptomatic treatments, long-term care, surveillance or monitoring, rehabilitation, palliative services, self-management, etc. Whereas clinical guidelines provide generic standards and recommendations, CPWs consider the local organization of services, available competencies and resources, healthcare provider structures, and care systems [112]. The application of every step of a CPW in any given patient may also depend on their disease presentation, severity, and psychosocial circumstances.

### Future perspectives

In this post-genome era, where we can generate huge amounts of molecular and other health data, we are rapidly entering the stage of scalable data interpretation of “-omic” technologies. To capture the power and promise of this health data revolution, we will rely exceedingly on algorithms to help us decipher the “data patterns” and identify useful ones as health biomarkers. We will further need to harmonize across-omics beyond genomes, extending from matchmaking carriership of rare genetic variants in patients with similar diseases to broadly assessing the sharing of multiple layers of -omics signatures. This omics and the broader health information revolution is inevitable as it follows similar data-driven transformation in other aspects of society, including communications, financial, transportation, social, and marketing industries, to name a few. With greater power comes greater responsibility; hence, it is critical to continue in parallel to develop the regulatory, ethical, and other societal standards to increase benefits and minimize risk and harm from these technologies.

There is an opportunity to build on existing technologies and informatic approaches that have been largely or frequently applied to diagnostics and extend these for treatment monitoring, be it through metabolic biomarkers, digital (e.g. imaging biomarkers), or epigenetic signatures of various stages of disease. To pick one example, phenotypic terminologies and ontologies have largely supported static cross-sectional implementations of individual terms. A key advance would be to systematically connect these terms into standardized longitudinal disease profiles that not only better support current, e.g. diagnostic use cases but can also support more precise and objective, personalized, predictive, preventative, and treatment measures.

There are multiple factors to address the need for better diversity and equity in diagnostics, and a detailed discussion of these is out of the scope of this review and has been discussed elsewhere [113]. One critical area to highlight is the emergence of the CARE Principles of Indigenous Data Governance, which provide an international framework for the ethical use of Indigenous data [114]. CARE Principles reflect the crucial role of culturally safe and responsive approaches supporting appropriate data acquisition, data use, and sharing, and complement the existing FAIR principles [115] as a key principle guiding equity and ethics in open data movements.

Once a diagnosis is established, the patient’s journey continues. And it must do so towards personalized care and therapeutic interventions to prevent morbidity and disability. Given the ever-growing number of RD interventions and ongoing trials, this can be a daunting task for the clinician. Indeed, several resources have been created to support the patient, family, and clinicians to get the therapies on the radar. Firstly, the ‘Treatabolome’, defined as a database of RD-specific treatments directly linked to the gene and variant level, will allow the flagging of already available therapies at the time of diagnosis [116]. Initially developed for neurometabolic (Treatable Intellectual disability app [2] and neurogenetic diseases (neuromuscular, epilepsy), this is now branching out to all phenotypes and conditions. Secondly, the UTOPIA (Unlocking Treatment Options, Personalized In-Time Access) RDs knowledge management platform, deployed at the Rare Care Centre at Perth Children’s Hospital, uses an individual’s phenotypic and (molecular) diagnostic information in combination with externally curated data sets (e.g. Orphanet, Clinicaltrials.gov, etc.) and AI. It delivers personalized healthcare summaries and pathways, and individualized flagging of relevant clinical trials, research, and non-health services (e.g. in education, community, and disability sectors). Ideally, treatment options are flagged in the ES/GS report for the clinician to consider lower thresholds and avoid delays.

New targets for treatment are identified at the genomic, epigenomic-transcriptomic, and metabolomic levels using model systems, such as (differentiated) iPSCs, organoids, and organisms. Deep phenotyping of these models allows for biomarker (e.g. metabolic, radiologic, epigenetic) identification. Potentially treatable manifestations can be identified via radiological, electrophysiological, hematological, somatic, neuropsychiatric, and contextual characterization. Reliable and relevant outcome measures are essential for adequately evaluating treatment safety and efficacy of novel interventions or repurposed drugs for RDs. Clinical heterogeneity and small patient numbers require special trial designs (e.g. N-of-1) and biostatistics. Personalized outcome measures patient & family participation with proper ethical and legal considerations are central in the process.

Technological advances drive the need for better education and workforce capacity building to ensure efficient, equitable, culturally appropriate, and value-adding deployment of existing and emerging diagnostics. This necessitates novel educational approaches that complement existing training paradigms that build capacity within primary care and specialist care, enable communities of practice between primary and specialist care (e.g. Project ECHO^®^), and are accessible and tailored to a diverse range of stakeholders and healthcare practitioners be they doctors, nurses, allied health, pharmacists or others.

## Conclusion

Adopting genomic testing technologies such as genome sequencing, transcriptomics, epigenomics, and functional genomic technologies such as metabolomics for RDs is expected to impact healthcare systems significantly. Genomic testing technologies offer several benefits, such as improved accuracy in diagnosis, personalized treatment plans, and the potential to develop new therapies. This information can then be used to develop personalized patient treatment plans, such as gene or targeted drug therapies. Furthermore, bioinformatics and AI can help healthcare professionals analyze large genomic datasets and identify patterns that may be difficult to detect through manual analysis. This can help identify new treatment targets and improve the efficiency and accuracy of RD diagnosis and treatment. The developments in genomic testing technologies, bioinformatics, and AI for healthcare are part of a broader trend toward using big data and AI in many areas of our society. In recent years, big data and AI have transformed many industries, including finance, transportation, retail, and entertainment.

Despite these benefits, implementing genomic testing technologies into healthcare systems presents several challenges. One significant challenge is the high cost of testing, which can limit accessibility for patients, particularly in low-income or resource-limited settings. For example, the cost of GS can range from a few hundred to several thousand dollars, making it difficult for some patients to afford [117–119]. To address this challenge, efforts are being made to develop more affordable and scalable genomic testing technologies. Recognition and classification of pathogenic genetic variation is incomplete but improving using technologies described here. Another challenge is the interpretation of genomic data, which requires specialized knowledge and expertise that may only be readily available in some healthcare settings. Training programs are being developed to address this challenge and increase the number of healthcare professionals with expertise in genomic testing interpretation.

Finally, implementing genomic testing technologies into healthcare systems will require changes in how healthcare is delivered, policies, and guidelines. For example, health systems will need to develop policies and guidelines to address ethical and legal issues related to genomic testing, such as patient confidentiality and genetic discrimination. Furthermore, healthcare providers will need to be educated about the benefits and limitations of genomic testing to ensure appropriate use and interpretation of the data.

In conclusion, adopting genomic testing technologies for RDs has the potential to transform healthcare systems. Despite the challenges associated with implementing these technologies, efforts are underway to develop more affordable and scalable genomic testing technologies, increase healthcare professionals’ expertise in genomic testing interpretation, and develop policies and guidelines to ensure ethical and appropriate use of genomic data. With continued efforts to overcome these challenges, integrating genomic testing into healthcare systems can revolutionize the diagnosis and treatment of RDs, benefiting patients and their families.

In relation to diagnosis, every time we make a diagnosis, we learn something new about the function of the genome that can contribute to developing therapies in the long term. Technological innovation to create effective and accessible diagnostic tools creates an important avenue to a healthier future for individuals living with RDs.

## Data Availability

Not applicable.

## Abbreviations

2D: 2-dimensional
3D: 3-dimensional
AI: Artificial intelligence
ALD: Adrenoleukodystrophy
ATAC-seq: Assay for transposase-accessible chromatin using sequencing
AVE: Atlas of variant effects
CARE: Collective benefit, authority to control, responsibility, and ethics
ClinGen: Clinical genome resource
CPWs: Care pathways
DNA: Deoxyribonucleic acid
DNAm: DNA methylation
ES: Exome sequencing
FAIR: Findability, accessibility, interoperability, and reusability
GA4GH: Global Alliance for Genomics and Health
GC: Gene curation
GDR: Gene-disease relationships
GenCC: Gene curation coalition
GS: Genome sequencing
GSEA: Gene set enrichment analysis
HPO: Human Phenotype Ontology
iPSCs: Induced pluripotent stem cells
IRDiRC: International Rare Diseases Research Consortium
KR: Knowledge representation
MAVEs: Multiplexed assays for variant effects
MME: Matchmaker exchange
NGS: Next-generation sequencing
OMIM: Online inheritance in man
RD: Rare diseases(s)
RNA: Ribonucleic acid
ROI: Return on investment
SNP: Single nucleotide polymorphism
SNV: Single nucleotide variant
UMLS: Unified medical language system
UTOPIA: Unlocking treatment options, personalised in-time access
UTR: UTR sequences
VUS: Variant of uncertain significance
WGS: Whole genome sequencing
